# Family Support and Modifiable Dementia Risk Factors Among Cognitively Impaired Older Adults in South Korea

**DOI:** 10.1093/geroni/igaf122.2920

**Published:** 2025-12-31

**Authors:** Minji Shim, Giyeon Kim

**Affiliations:** Chung-Ang University, Seoul, Republic of Korea; Chung-Ang University, Seoul, Republic of Korea

## Abstract

With a rapidly growing aging population, cognitive impairment is a pressing public health issue, and the importance of family support is growing. This study investigates the relationship between family support and modifiable dementia risk factors among older adults with cognitive impairment in South Korea. Family support was categorized by the presence of a spouse, children, and co-residence with children. For risk factor selection, we focused on three key modifiable dementia risk factors identified by the Lancet Commission: smoking, depressive symptoms, and social isolation. We analyzed data from the 2023 Korean National Living Survey for older adults with cognitive impairment(n = 4,009). Binomial logistic regression was conducted to examine the association between these family support categories and three modifiable dementia risk factors. The results indicated significant associations between family support and dementia risk factors. Older adults without a spouse and children were more likely to experience social isolation (OR = 1.51, p<.001) and depressive symptoms (OR = 1.35, p < 0.001) compared to those with a spouse and co-residing children. However, smoking was not significantly associated with family support. These findings highlight the protective role of family support in mitigating modifiable dementia risk factors among cognitively impaired older adults. Given the evolving care culture in Korea due to modernization and nuclear family structures, policies and interventions that strengthen family and social support networks may help reduce these risk factors and improve overall well-being in this population.

